# Glucosylated cholesterol in mammalian cells and tissues: formation and degradation by multiple cellular β-glucosidases[S]

**DOI:** 10.1194/jlr.M064923

**Published:** 2016-03

**Authors:** André R. A. Marques, Mina Mirzaian, Hisako Akiyama, Patrick Wisse, Maria J. Ferraz, Paulo Gaspar, Karen Ghauharali-van der Vlugt, Rianne Meijer, Pilar Giraldo, Pilar Alfonso, Pilar Irún, Maria Dahl, Stefan Karlsson, Elena V. Pavlova, Timothy M. Cox, Saskia Scheij, Marri Verhoek, Roelof Ottenhoff, Cindy P. A. A. van Roomen, Navraj S. Pannu, Marco van Eijk, Nick Dekker, Rolf G. Boot, Herman S. Overkleeft, Edward Blommaart, Yoshio Hirabayashi, Johannes M. Aerts

**Affiliations:** Department of Medical Biochemistry,* Academic Medical Center, Amsterdam, The Netherlands; Departments of Medical Biochemistry† Leiden Institute of Chemistry, Leiden, The Netherlands; Bio-organic Synthesis,** Leiden Institute of Chemistry, Leiden, The Netherlands; Brain Science Institute,§ RIKEN, Wako, Japan; Centro de Investigación Biomédica en Red de Enfermedades Raras,†† Unidad de Investigación Traslacional, Zaragoza, Spain; Department of Molecular Medicine and Gene Therapy,§§ Lund University, Lund, Sweden; Addenbrooke’s Hospital,*** Department of Medicine, University of Cambridge, Cambridge, UK

**Keywords:** glucosyl-β-D-cholesterol, glucosylceramide, glucocerebrosidase, Gaucher disease, Niemann-Pick type C disease

## Abstract

The membrane lipid glucosylceramide (GlcCer) is continuously formed and degraded. Cells express two GlcCer-degrading β-glucosidases, glucocerebrosidase (GBA) and GBA2, located in and outside the lysosome, respectively. Here we demonstrate that through transglucosylation both GBA and GBA2 are able to catalyze in vitro the transfer of glucosyl-moieties from GlcCer to cholesterol, and vice versa. Furthermore, the natural occurrence of 1-*O*-cholesteryl-β-D-glucopyranoside (GlcChol) in mouse tissues and human plasma is demonstrated using LC-MS/MS and ^13^C_6_-labeled GlcChol as internal standard. In cells, the inhibition of GBA increases GlcChol, whereas inhibition of GBA2 decreases glucosylated sterol. Similarly, in GBA2-deficient mice, GlcChol is reduced. Depletion of GlcCer by inhibition of GlcCer synthase decreases GlcChol in cells and likewise in plasma of inhibitor-treated Gaucher disease patients. In tissues of mice with Niemann-Pick type C disease, a condition characterized by intralysosomal accumulation of cholesterol, marked elevations in GlcChol occur as well. When lysosomal accumulation of cholesterol is induced in cultured cells, GlcChol is formed via lysosomal GBA. This illustrates that reversible transglucosylation reactions are highly dependent on local availability of suitable acceptors. In conclusion, mammalian tissues contain GlcChol formed by transglucosylation through β-glucosidases using GlcCer as donor. Our findings reveal a novel metabolic function for GlcCer.

Membranes of higher eukaryotic cells contain glycerolipids, sterols, and sphingolipids. For each of these lipid classes, monoglucosylated structures have been reported. Glucosylceramide (GlcCer), the intermediate in biosynthesis and degradation of more complex glycosphingolipids (GSLs), is ubiquitous in mammalian cells, particularly located in the cell membrane (1). Its presence in plants and some fungi is also documented. Glucosyldiacylglycerol has been identified in various plants, but its presence in mammalian cells is comparatively poorly documented (2, 3). Likewise, sterol-glucosides are known to occur in plants and fungal species (4), but their existence in mammalian cells has not been extensively studied. Indications of the existence of glucosyl-β-D-cholesterol or 1-*O*-cholesteryl-β-D-glucopyranoside (GlcChol) in mammalian cells were first provided by Murofushi and coworkers (5, 6). They described its occurrence in cultured human fibroblasts and gastric mucosa (5, 6). Heat shock was found to increase biosynthesis of GlcChol and, subsequently, induce HSP70 (7). GlcCer is formed by the enzyme GlcCer synthase (GCS, EC2.4.1.80). This transferase, first cloned by Hirabayashi and colleagues (8), is located at the cytosolic leaflet of the Golgi apparatus where it transfers the glucose-moiety from UDP-glucose to ceramide (9). In a recent study, Akiyama et al. (10) showed that GCS does not synthesize GlcChol. They noticed that GM-95 cells deficient in GCS are unable to synthesize GlcChol without the addition of exogenous GlcCer. Furthermore, the same researchers demonstrated that, at least in vitro, the lysosomal enzyme glucocerebrosidase (GBA; E.C.3.2.1.45) generates, through transglucosylation, 25-[*N*-[(7-nitro-2-1,3-benzoxadiazol-4-yl) methyl] amino]-27-norcholesterol (25-NBD-cholesterol)-glucoside from GlcCer and artificial 25-NBD-cholesterol (11). Such ability of GBA to perform transglucosylation was earlier demonstrated by Glew and coworkers, showing catalyzed transfer of the glucose moiety from 4-methylumbelliferyl-β-glucoside to retinol and other alcohols (12).

The enzyme GBA is well-studied because its deficiency underlies Gaucher disease (GD), a relatively common lysosomal storage disease (13). Assisted by the small activator protein, saposin C, GBA degrades GlcCer to ceramide and glucose in lysosomes, the penultimate step in GSL catabolism (13). Deficient GBA activity in GD patients consequently results in accumulation of GlcCer in lysosomes, most prominently in macrophages. These “Gaucher cells” secrete specific proteins, as well as glucosylsphingosine (GlcSph), the deacylated form of GlcCer (14–16). The non-neuronopathic (type 1) variant of GD is presently treated by enzyme replacement therapy (ERT), implying chronic twice-weekly intravenous infusion of macrophage-targeted recombinant enzyme (17). An alternative treatment of type 1 GD, named substrate reduction, is based on oral administration of an inhibitor of GCS (18–20).

Mammalian cells and tissues contain other β-glucosidases besides GBA that degrade GlcCer. All cells express the membrane-associated nonlysosomal glucosylceramidase, named GBA2 (21–23). This enzyme is not deficient in GD patients. In fact, a compensatory overexpression of GBA2 in materials of GD has been reported (24). GBA2 has been found to be located outside lysosomes, being noted at the endoplasmic reticulum in hepatocytes (22), at the endoplasmic reticulum and Golgi apparatus in HEK293 cells overexpressing enzyme (25), and at the endosomes in fibroblasts and COS-7 cells (23). GBA2 degrades GlcCer without need for an activator protein, and further differs from GBA in noted artificial substrate and inhibitor specificity (21). Finally, some tissues express the enzyme GBA3, also referred to as broad-specific cytosolic β-glucosidase (26). This enzyme shows relatively poor in vitro hydrolytic activity toward GlcCer and is thought to be primarily involved in detoxification of glucosylated xenobiotics (26). All three human retaining β-glucosidases employ the double displacement mechanism in catalysis. There are many documented examples of transglucosylation mediated by retaining glycosidases (27). Therefore, theoretically, in addition to GBA, GBA2 and GBA3 might also generate GlcChol.

Modification of cholesterol by glucosylation changes the physicochemical properties of the sterol, rendering it far more water soluble. Given the potential physiological relevance, the natural occurrence of GlcChol and its metabolism in cells and tissues are of interest. We therefore studied the existence of the glucosylated sterol in mammalian tissues. For this purpose ^13^C_6_-isotope-labeled GlcChol was synthesized to be used as internal standard in sensitive quantitative detection of GlcChol by LC-MS/MS. Here we demonstrate the natural occurrence of GlcChol in mammalian cells and tissues. Moreover, we document the ability of both GBA and GBA2 to degrade, as well as synthesize, GlcChol. The importance of substrate and acceptor concentrations regarding the action of GBA and GBA2 in GlcChol metabolism is experimentally demonstrated. Our investigation demonstrates the surprising versatility of β-glucosidases, a finding discussed in relation to metabolism of sphingolipids and sterols in health and disease.

## MATERIALS AND METHODS

### Materials

The 25-NBD-cholesterol, *N*-[6-[(7-nitro-2-1,3-benzoxadiazol-4-yl)amino]hexanoyl]D-glucosyl-β1-1′-sphingosine (C6-NBD-GlcCer), *N*-[6-[(7-nitro-2-1,3-benzoxadiazol-4-yl)amino]hexanoyl]D-erythro-sphingosine (C6-NBD-Cer), D-glucosyl-β-1,1′ *N*-palmitoyl-D-*erythro*-sphingosine (C16:0-GlcCer), and D-glucosyl-β-1,1′*N*-oleoyl-D-*erythro*-sphingosine (C18:1-GlcCer) were purchased from Avanti Polar Lipids (Alabaster, AL). The 4-methylumbelliferyl β-D-glucopyranoside (4MU-Glc) was purchased from Glycosynth™ (Winwick Quay Warrington, Cheshire, UK). Conduritol B epoxide (CBE; D,L-1,2-anhydro-*myo*-inositol;) was from Enzo Life Sciences Inc. (Farmingdale, NY), GlcChol (β-cholesteryl glucoside, β-GlcChol) and ammonium formate (LC-MS quality) were from Sigma-Aldrich (St. Louis, MO). *N*-(*n*-butyl)deoxygalactonojirimycin (NB-DGJ) was purchased from Toronto Research Chemicals (Toronto, Canada). GBA2 inhibitor, *N*-(5-adamantane-1-yl-methoxy-pentyl)-deoxynojirimycin (AMP-DNM), and GBA3 inhi­bitor, α-1-C-nonyl-DIX (anDIX), were chemically synthesized in the department of Bio-organic Synthesis at the Faculty of Science, Leiden Institute of Chemistry at the University of Leiden (Leiden, The Netherlands). Cerezyme^®^, a recombinant human GBA (rGBA), was obtained from Genzyme (Genzyme Nederland, Naarden, The Netherlands). Cholesterol trafficking inhibitor, U18666A, and methyl-β-cyclodextrin were from Sigma-Aldrich Chemie GmbH. LC-MS-grade methanol, 2-propanol, water, and HPLC-grade chloroform were purchased from Biosolve, and ammonium formate (LC-MS grade) from Sigma-Aldrich Chemie GmbH.

### Synthesis of ^13^C_6_ isotope-labeled β-cholesteryl glucoside

The synthesis of ^13^C-labeled glucosyl donor **4** (see **Scheme 1**) commences with protecting the five hydroxyls in glucose **1** as the benzoyl esters using pyridine and benzoyl chloride to give 1,2,3,4,6-penta-*O*-benzoyl-β-D-^13^C_6_-glucopyranoside **2** quantitatively. In the next step the anomeric benzoate was selectively removed using hydrazine acetate providing 2,3,4,6-tetra-*O*-benzoyl-α/β-D-^13^C_6_-glucopyranoside **3** in 82% yield. The anomeric hydroxyl in **3** was transformed into the corresponding trichloroacetimidate using trichloroacetonitrile and 1,8-diazabicyclo[5.4.0]undec-7-ene as base, giving 2,3,4,6-tetra-*O*-benzoyl-1-(2,2,2-trichloroethanimidate)-α-D-^13^C_6_-glucopyranoside **4**. In the penultimate step, cholesterol was reacted with **4** under the agency of a catalytic amount of trimethylsilylmethanesulphonate in dichloromethane at room temperature. After 1 h, the reaction was quenched with triethylamine and the mixture purified by silica gel column chromatography giving cholesteryl 2,3,4,6-tetra-*O*-benzoyl-β-D-^13^C_6_-glucopyranoside **5** in 83%. Compound **5** was deprotected using sodium methoxide in methanol/dichloromethane giving, after silica gel column chromatography, the title compound, cholesteryl-β-D-^13^C_6_-glucopyranoside (^13^C_6_-GlcChol) **6** as a white solid in 94%.

Scheme 1.Synthesis of ^13^C_6_-GlcChol **6** (28).
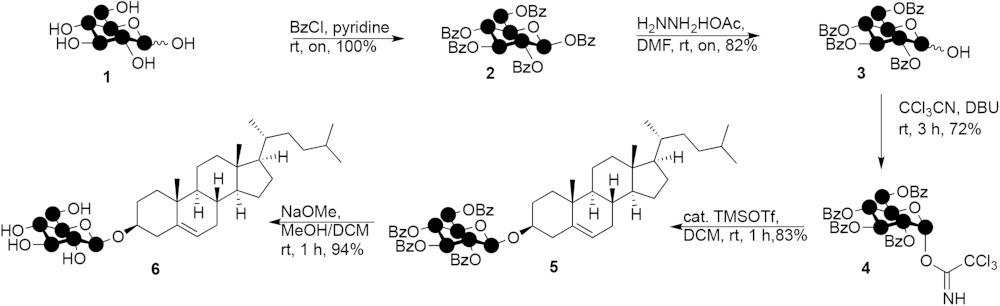


### Animal studies

*Npc1*^−/−^ mice (*Npc1*^nih^ and *Npc1*^spm^), along with WT littermates (*Npc1*^+/+^), were generated by crossing *Npc1*^+/−^ males and females in-house. The heterozygous BALB/c Nctr-*Npc1^m1N^*/J mice (stock number 003092) and heterozygous C57BLKS/J-*Npc1^spm^*/J (stock number 002760) were obtained from the Jackson Laboratory (Bar Harbor, ME). Mouse pups were genotyped according to published protocols (29, 30). The *Gba2*^−/−^ mice (C57Bl/6-129S6/SvEv mixed background) were generated as previously described (22). Breeding pairs of LIMP-2 were kindly provided by Prof. Paul Saftig (Kiel, Germany) (31). Homozygous WT animals (LIMP2^+/+^) and homozygous animals (LIMP2^−/−^) were generated by crossing heterozygous (LIMP2^+/−^) mice. Genotyping was determined by PCR using genomic DNA (31). Mice (±3 weeks old) received the rodent AM-II diet (Arie Blok Diervoeders, Woerden, The Netherlands). The mice were housed at the Institute Animal Core Facility in a temperature- and humidity-controlled room with a 12 h light/dark cycle and given access to food and water ad libitum. All animal protocols were approved by the Institutional Animal Welfare Committee of the Academic Medical Centre Amsterdam in the Netherlands (DBC101698, DBC100757-115, DBC100757-125, and DBC17AC)

The generation of the GD1 mouse model has been described previously (32, 33). Mice were maintained in individually ventilated cages with ad libitum food and water in the animal facility at Lund University Biomedical Center. Breeding and experimental procedures were approved by the Committee for Animal Ethics in Malmö/Lund, Sweden.

Animals were first anesthetized with a dose of Hypnorm (0.315 mg/ml phenyl citrate and 10 mg/ml fluanisone) and Dormicum (5 mg/ml midazolam) according to their weight. The given dose was 80 μl/10 g bodyweight. Animals were euthanized by cervical dislocation. Organs were collected by surgery, rinsed with PBS, directly snap-frozen in liquid nitrogen, and stored at −80°C. Later, homogenates were made from the frozen material in 25 mM potassium phosphate buffer (pH 6.5), supplemented with 0.1% (v/v) Triton X-100 and protease inhibitors (4 μl of buffer per 1 mg of tissue).

### Cloning of cDNAs encoding GBA2, GBA3, and UGCG

The design of cloning primers was based on NCBI reference sequences NM_172692.3 for murine GBA2, NM_020973.3 for human hGBA3, and NM_003358.2 for human UGCG (GCS). Using the primers listed below, the full-length coding sequences were cloned into pcDNA3.1/Myc-His (Invitrogen, Life Technologies, Carlsbad, CA), using primers: *RB143*, GAATTCGCCGCCACC­ATGGTAACCTGCGTCCCGG and *RB144*, GCGGCCGCTCTG­AATTGAGGTTTGCCAG for mGBA2; *RB252*, GAATTCGCCGCCACCATGGCTTTCCCTGCAGGATTTG and *RB253*, GCGGCCGCTACAGATGTGCTTCAAGGCC for hGBA3; *RB111*, TCCTGCGGGAGCGTTGTC and *RB114*, GGTACCTACATCTAGGA­TTTCCTCTGC for hUCGC. These constructs were used to transfect COS-7 cells.

For the transfection of Chinese hamster ovary cells (CHO-K1 cells), the full-length coding sequence for transcript variant 1 of human GBA3 (NM_020973.3) was cloned into p3xFLAG-CMV-14 (Sigma-Aldrich) as described previously (11).

### Cell culture and transfection

RAW264.7 cells were obtained from the American Type Culture Collection and were cultured in DMEM (Life Technologies, Carlsbad, CA) supplemented with 10% FBS (Bodinco, Alkmaar, The Netherlands) with penicillin/streptomycin (Life Technologies). COS-7 cells were cultured in Iscove’s modified Dulbecco’s medium with 5% FBS and penicillin/streptomycin under 5% CO_2_ at 37°C. Cells were seeded at 75% confluence in 6-well plates and transfected using FuGENE^®^ 6 transfection reagent (Promega Benelux, Leiden, The Netherlands) according to the manufacturer’s instructions, at a FuGENE:DNA ratio of 3:1. After 24 h, inhibitors of GBA (CBE, 300 μM) or GBA2 (AMP-DNM, 20 nM) were added and 48 h later, the medium was removed, cells were washed three times with ice-cold PBS and harvested by scraping in 25 mM potassium-phosphate buffer (pH 6.5). CHO-K1 cells (RCB0285, established by T. T. Puck) were purchased from RIKEN BioResource Center (Ibaraki, Japan) and cultured in Ham’s F-12 medium (Nissui) supplemented with 10% FBS under 5% CO_2_ at 37°C. cDNA transfection for CHO-K1 cells was carried out using Lipofectamine^®^ 2000 transfection reagent (Life Technologies) according to the manufacturer’s instructions. After 24 h, medium containing transfection reagents was removed and cells were incubated with lysis buffer [50 mM Tris-HCl, 150 mM NaCl, 1 mM EDTA, 1% Triton X-100, 1 tablet/10 ml Complete Protease Inhibitor Cocktail Tablets (Roche, Basel, Switzerland) (pH 7.4)] for 15–30 min at 4°C after washing with PBS. The cells were harvested and centrifuged at 12,000 *g* for 10 min at 4°C. The obtained supernatants were collected for in vitro enzyme assays.

### In vitro assay of transglucosylase activity

Lysates of COS-7 cells overexpressing GBA2, GBA3, GCS, and rGBA were used to determine transglucosylase activity of each enzyme. The assay was performed as described earlier (11) with a few modifications. First, 40 μl of homogenate of cells overexpressing GBA2, GBA3, or GCS was preincubated with 10 μl of 25 mM CBE in water for 20 min (samples containing diluted rGBA were preincubated in the absence of CBE). To each of the samples, 200 μl of the appropriate buffer containing 100 μM of donor (either C18:1-GlcCer or GlcChol) and 40 μM of acceptor (either 25-NBD-cholesterol or C6-NBD-Cer) were added. Transglucosylase activity of GBA2-overexpressing cells was measured in a 150 mM McIlvaine buffer (pH 5.8) and the assay for rGBA was done in a 150 mM McIlvaine buffer (pH 5.2) containing 0.1% BSA, 0.1% Triton X-100, and 0.2% sodium taurocholate. For GBA3, the assay contained 100 mM HEPES buffer (pH 7.0). The transglucosylase assay for GCS was performed in a 125 mM potassium-phosphate buffer (pH 7.5) with 12.5 mM UDP-glucose, 6.25 mM MgCl_2_, 0.125% BSA, and 0.625% CHAPS. After 1 h of incubation at 37°C, the reaction was terminated by addition of chloroform/methanol (1:1, v/v) and lipids were extracted according to Bligh and Dyer (34). Thereafter, lipids were separated by TLC on HPTLC silica gel 60 plates (Merck, Darmstadt, Germany) using chloroform/methanol (85:15, v/v) as eluent followed by detection of NBD-labeled lipids using a Typhoon variable mode imager (GE Healthcare Bio-Science Corp., Piscataway, NJ) (35).

Identification of newly formed fluorescent lipid in transglucosylation assays with 25-NBD-cholesterol as acceptor was performed following its isolation by scraping from plates by demonstration of complete digestion to NBD-cholesterol using excess rGBA at pH 5.2 (McIlvaine buffer) in the presence of 0.2% (w/v) sodium taurocholate and 0.1% (v/v) Triton X-100.

Lysates of CHO-K1 cells were used to access the transglucosylase activity and the β-glucosidase activity of GBA3. The assay for transglucosylase activity was performed according to the method we established previously (11) with slight modifications. The reaction mixture, in a total volume of 20 μl, contained 40 μM 25-NBD-cholesterol, 80 μM C16:0-GlcCer, 50 mM citrate-phosphate buffer (pH 6.2), 0.5% CHAPS, 2% ethanol, and the desired amount of enzyme. After incubation at 37°C for 20 h, the reaction was terminated by adding chloroform/methanol (2:1, v/v), and the lipids were extracted and analyzed as reported before (11). The assay for β-glucosidase activity was performed according to the method we established previously (11) with slight modifications. The reaction mixture, in a total volume of 20 μl, contained 100 pmol C6-NBD-GlcCer, 50 mM citrate-phosphate buffer (pH 6.2), and a desired amount of enzyme. After incubation at 37°C for 30 min, the reaction was terminated by adding chloroform/methanol (2:1, v/v), and the lipids were extracted and analyzed as reported before (11).

### Analysis of GlcChol by LC-MS/MS

**Fig. 1.**
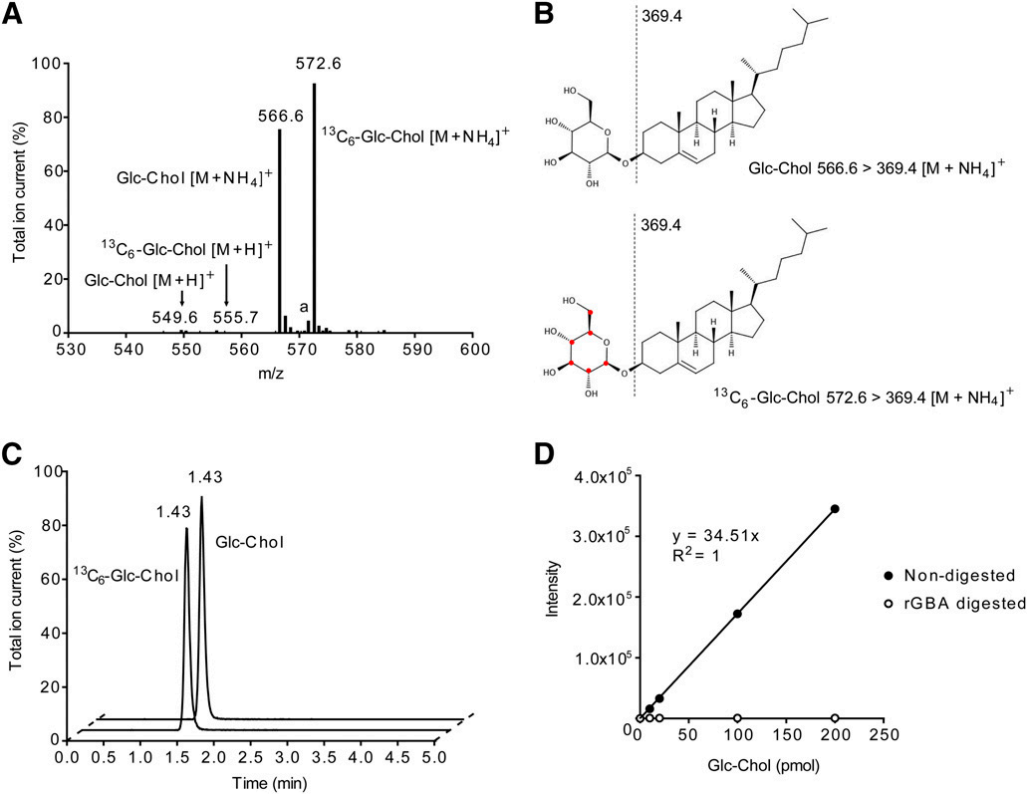
Quantification of GlcChol by LC-MS/MS. A: MS scan of pure GlcChol and its ^13^C-labeled isotope. The ammonium-adduct is the most abundant *m/z* for both compounds. The product ion, *m/z* 369.4, is the common fragment for both compounds. Shown are the parent scans of product ion *m/z* 369.4 of GlcChol and ^13^C_6_-labeled GlcChol, [M+NH^4^]^+^, *m/z* 566.6 for GlcChol, and *m/z* 572.6 for ^13^C_6_-GlcChol. The [M+H]^+^ and [M+Na]^+^ are the minor *m/z*. The *m/z* 571.6 represents the sodium adduct of GlcChol (a). B: The structure of GlcChol and its isotope, ^13^C_6_-labeled GlcChol, their fragmentation pattern, *m/z* 369.4, is the product ion of both compounds after loss of glucose moiety. C: Elution pattern of GlcChol (*m/z* 566.6 > 369.4) and ^13^C_6_-labeled GlcChol (*m/z* 572.6 > 369.4) from UPLC. D: Linearity of GlcChol quantification and its complete digestion with rGBA (1.0 U/ml) for 18 h at 37°C.

A Waters Acquity^TM^ TQD instrument was used in all experiments. The instrument consisted of a UPLC system combined with a tandem quadrupole mass spectrometer as mass analyzer. Data were analyzed with Masslynx 4.1 software (Waters Corporation, Milford, MA). GlcChol and ^13^C_6_-GlcChol (internal standard) were separated using a BEH C18 reversed-phase column (2.1 × 50 mm, particle size 1.7 μm; Waters Corporation), by applying an isocratic elution of mobile phases, 2-propanol:water 90:10 (v/v) containing 10 mM ammonium formate (eluent A) and methanol containing 10 mM ammonium formate (eluent B). The UPLC program was applied during 5 min consisting of 10% A and 90% B. The divert valve of the mass spectrometer was programmed to discard the UPLC effluent before (0–0.25 min) and after (4–5 min) the elution of the analytes to prevent system contamination. The flow rate was 0.250 ml/min and the retention time of both GlcChol and the internal standard was 1.43 min (Fig. 1C). The column temperature and the temperature of the autosampler were kept at 23°C and 10°C, respectively, during the run.

Solutions of GlcChol and ^13^C_6_-GlcChol and a mixture of both compounds were prepared with concentrations of 1 pmol/μl in 5 mM ammonium formate in methanol. The compounds were introduced in the mass spectrometer by direct infusion and the optimal tuning conditions for both compounds in ES^+^ (electrospray positive) mode were determined (**Table 1**). The most abundant species for both compounds were ammonium adducts, [M+NH_4_]^+^, *m/z* 566.6 > 369.4 for GlcChol and *m/z* 572.6 > 369.4 for ^13^C_6_-GlcChol (see also Fig. 1B). The product ion represents the cholesterol part of the molecule after loss of the glucose moiety. Because the ^13^C isotopes are on the glucose molecule, the daughter fragment of ^13^C_6_-GlcChol has the same *m/z* ratio of 369.4.

**TABLE 1.** MS/MS instrument parameters

Capillary voltage	3.50 KV
Cone voltage	20 V
Source temperature	120°C
Desolvation temperature	450°C
Cone gas	50 l/h
Desolvation gas	950 l/h
Collision gas	0.20 ml/min
Collision voltage	20 V
Type	Multiple reaction monitoring
Ion mode	ES^+^ (electrospray positive)
Dwell time	0.250 s
Interchannel delay	0.005 s
Interscan delay	0.005 s
Transitions	
GlcChol, RT (min)	1.43
^13^C_6_-GlcChol, RT (min)	1.43
Fit weight, RT (min)	None
Smooth method, RT (min)	Mean
Smooth width, RT (min)	2

RT, retention time.

Confirmation of compounds with *m/z* 566.6 > 369.4 being GlcChol was performed by demonstration of complete digestion to cholesterol using excess rGBA at pH 5.2 (McIlvaine buffer). The release of glucose was confirmed by a glucose oxidase assay.

### Collection of Niemann-Pick type C and GD patient plasma

EDTA plasma (15 males and 3 females) was collected prior to therapy from Dutch patients suffering from type 1 GD, known by referral to the Academic Medical Center. Diagnosis of GD in patients was confirmed by genotyping and demonstration of deficient GBA activity in leukocytes or fibroblasts. Plasma samples were stored frozen at −20°C until further use. EDTA plasma samples of 42 male and 47 female control subjects were collected at the Academic Medical Center. The EDTA plasma samples of 15 Niemann-Pick type C (NPC) patients and 9 NPC carriers were collected at the Unidad de Investigación Traslacional in Zaragoza, Spain.

The status of affected or carrier of NPC disease was determined after the exomic sequencing of *NPC1* and *NPC2* genes, according to the presence of two mutations or one mutation, respectively. Filipin stainings of fibroblasts were conducted to complete the diagnosis study. Plasma samples were stored frozen at −20°C until further use. Approval had been obtained from the institutional ethics committee and informed consent according to the Declaration of Helsinki.

### Quantification of total GlcChol in human plasma

For quantitative analysis of GlcChol in samples of plasma, we developed a LC-MS/MS method using the multiple reaction monitoring (MRM) mode of the transitions mentioned above. First, GlcChol was extracted from plasma from a healthy individual according the method of Bligh and Dyer (34) with a few modifications. Plasma (20 μl) was pipetted in an Eppendorf tube (2 ml) and 20 μl of an internal standard solution, containing 0.1 pmol/μl of ^13^C_6_-GlcChol in methanol, was added, followed by addition of 280 μl methanol and 150 μl of chloroform. After brief mixing, the sample was left at room temperature for 30 min, mixed occasionally, and centrifuged for 10 min at 15,700 *g* to spin down precipitated protein. The supernatant was transferred to an Eppendorf tube and 150 μl chloroform and 250 μl water were added to induce separation of phases. After centrifugation (5 min at 15,700 *g*), the lower hydrophobic phase was transferred to a clean Eppendorf tube and the upper phase was washed by addition of 300 μl of chloroform. Lower phases were pooled and taken to dryness at 35°C under a nitrogen stream. Next, the residue was dissolved in 700 μl of butanol and 700 μl of water, mixed well, and centrifuged for 10 min at 15,700 *g*. The upper phase (butanol) was transferred to a 1 ml tube with screw cap and the sample was dried under a gentle stream of nitrogen at 35°C. Subsequently, the residue was dissolved in 150 μl of eluent B by mixing and sonication, and after centrifugation (5 min at 15,700 *g*), an aliquot of 100 μl was transferred into an UPLC vial with insert. Ten microliters of the solution were injected for analysis.

Second, for the quantification of GlcChol in plasma, the sample was spiked with GlcChol (concentrations: 0–2.5–5–10–50–100–200–1,000 pmol GlcChol/ml of plasma), internal standard was added and samples were extracted. The ratio, the area from transition GlcChol over the area from the transition ^13^C_6_-GlcChol, was plotted against the concentration of GlcChol spiked in the plasma samples. A linear response was obtained over the entire concentration range (y = 0.0108x + 1.9188, R^2^ = 0.998). The within-run variation (164.2 ± 4.3 pmol/ml with coefficient of variation % 2.6) and between run variation (166.8 ± 3.6 pmol/ml with coefficient of variation % 2.2), was determined in plasma of a healthy volunteer by 10 repetitive measurements.

The limit of detection was 0.5 pmol/ml plasma with a signal-to-noise ratio of three and the limit of quantitation was 0.9 pmol/ml plasma with a signal-to-noise ratio of 10. Calculation of the signal-to-noise ratio was done using the peak-to-peak method.

### Analysis of GlcChol in animal tissues by LC-MS/MS

^13^C_6_-labeled GlcChol (3 pmol) in methanol was added to 30 μl of mouse tissue homogenate. Next, lipids were extracted according to the method of Bligh and Dyer (34) and GlcChol was analyzed by LC-MS, as described above.

### Analysis of GlcChol in COS-7 cells by LC-MS/MS

COS-7 cells overexpressing GBA2 or GCS were homogenized by sonication on ice. Prior to extraction, 2 pmol of ^13^C_6_-labeled GlcChol in methanol (used as an internal standard) was added to 180 μl of homogenate. Next, lipids were extracted according to the method of Bligh and Dyer (34) by addition of methanol, chloroform, and water (1:1:0.9, v/v/v) and the lower phase was taken to dryness under a stream of nitrogen. Isolated lipids were purified by water/butanol extraction (1:1, v/v) and GlcChol was analyzed by LC-MS, as described above.

### Analysis of GlcCer and ceramide in COS-7 cells by HPLC

COS-7 cells, overexpressing GBA2 or GCS, were homogenized by sonication on ice. Prior to extraction, 1 nmol of C17-sphinganine in methanol (used as an internal standard) was added to 100 μl of homogenate. Next, lipids were extracted according to the method of Bligh and Dyer (34) by addition of methanol, chloroform, and water (1:1:0.9, v/v/v) and the lower phase taken to dryness under a stream of nitrogen. Isolated lipids were deacylated in a microwave oven, derivatized, and analyzed by HPLC, as described before (36).

### Analysis of GlcChol in RAW264.7 cells by LC-MS/MS

^13^C_6_-labeled GlcChol (3 pmol) in methanol was added to 100 μl of RAW264.7 cell lysate. Next, lipids were extracted according to the method of Bligh and Dyer (34) and GlcChol was analyzed by LC-MS, as described above.

### Protein concentration

Protein concentration was determined using the Pierce BCA protein assay kit (Thermo Scientific) by the microplate procedure. Absorbance was measured in an EL808 Ultra microplate reader (BIO-TEK Instruments Inc.) at 550 nm.

### Statistical analysis

Values in the figures are presented as a mean ± SD. Data were analyzed by unpaired Student’s *t*-test or Mann-Whitney U test. *P* values <0.05 were considered significant (*P < 0.05, **P < 0.01, and ***P < 0.001).

## RESULTS

### Quantification of GlcChol by LC-MS/MS

To establish whether glucosyl-β-D-cholesterol (GlcChol) physiologically occurs in mammals, we first developed a LC-MS/MS procedure for its quantitative detection. For this purpose, a ^13^C_6_ isotope-labeled GlcChol was synthesized (^13^C_6_-GlcChol). The use of the isotope-labeled compound as internal standard avoids the need for corrections for extraction efficiency, chromatographic behavior, and ionization efficiency during quantification of GlcChol. To prevent undesired adduct formation, lipids were extracted in the absence of additional salts. To stimulate formation of desired ammonium adduct, we incorporated 10 mM ammonium in the eluent.

Sensitive quantitative measurement of GlcChol proved feasible with ^13^C_6_ isotope-labeled GlcChol as internal standard, as shown in **Fig. 1A–C**. The limit of detection was 0.5 pmol/ml plasma, with a signal-to-noise ratio of three and a limit of quantitation of 0.9 pmol/ml plasma with a signal-to-noise ratio of 10. GlcChol was found to be an excellent substrate for rGBA (Cerezyme^®^), even at sub-optimal conditions (absence of Triton X-100 and sodium taurocholate) (Fig. 1D).

### Demonstration of natural occurrence of GlcChol in mice

**Fig. 2.**
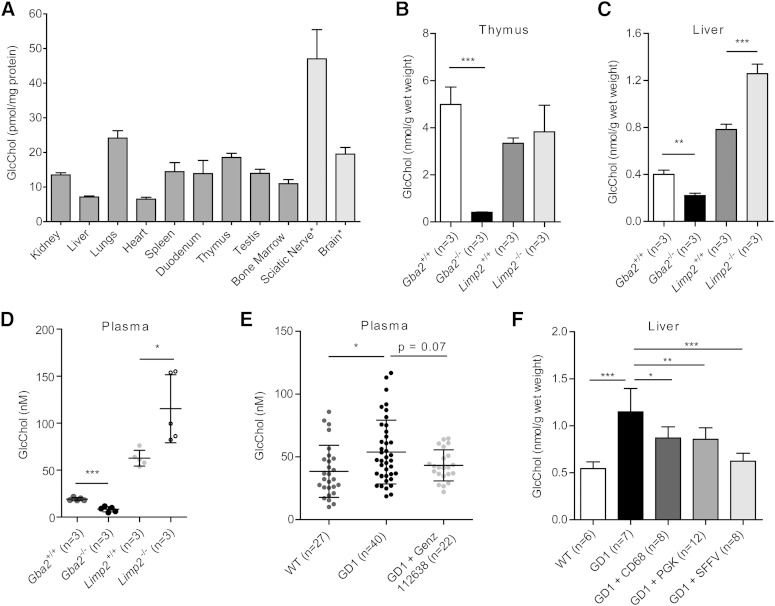
GlcChol in tissues of WT, GBA-deficient, and GBA2-deficient mice. A: GlcChol (picomoles per milligram protein) in various tissues of WT male mice at 2 months of age (n = 3, mean ± SD). *A significant proportion of *m/z* 566.6 was not digestible by rGBA. B: GlcChol (nanomoles per gram wet weight) in thymus of WT, *Gba2*^−/−^, and *Limp2*^−/−^ male mice at 2 months of age (n = 3, mean ± SD). C: GlcChol (nanomoles per gram wet weight) in liver of WT, *Gba2*^−/−^, and *Limp2*^−/−^ male mice at 2 months of age (n = 3, mean ± SD). D: GlcChol (nanomolar) in plasma of WT, *Gba2*^−/−^, and *Limp2*^−/−^ male mice at 2 months of age (n = 3, mean ± SD). E: Plasma GlcChol (nanomolar) in WT (n = 27), untreated type 1 GD mice (n = 40), and treated with Genz-112638 (n = 27) (mean ± SD). F: Liver GlcChol (nanomoles per gram wet weight) in WT mice (n = 6, mean ± SD), type 1 GD-induced mice untreated (n = 6), type 1 GD mice treated with lentiviral GBA cDNA gene therapy with macrophage-specific promotor (CD68) (n = 8), ubiquitously expressed human phosphoglycerate kinase (PGK) promotor (n = 12), or γretroviral vector with the viral promoter spleen focus forming virus (SFFV) promotor (n = 8). Data were analyzed using an unpaired *t*-test. *P < 0.05, **P < 0.01, and ***P < 0.001.

Next, we examined various tissues of WT mice on the presence of GlcChol. Relative high amounts of glucosylated sterol were noted for thymus, sciatic nerve, brain, and lungs (see **Fig. 2A**). The identity of the quantified structure (*m/z* 566.6) in thymus (and other tissues) was confirmed by its digestion by rGBA, showing that in WT animals more than 90% of the lipid measured is indeed glucosyl-β-D-cholesterol. However, in sciatic nerve and brain, a significant proportion of *m/z* 566.6 was not digested by rGBA, suggesting that it may represent another glycosylated cholesterol, for example galactosyl-β-D-cholesterol.

The concentration of GlcChol in liver and thymus was determined for tissues of WT mice, animals lacking GBA2 (22) and LIMP-2 KO mice with markedly reduced GBA due to impaired transport to lysosomes (31). As shown in Fig. 2B–D, the GlcChol concentration was markedly lower in all the tissues of GBA2-deficient animals analyzed compared with WT controls, especially in thymus. In contrast, in the GBA-deficient LIMP-2 KO mice, no reduction in GlcChol, but rather a significant increase in liver and plasma levels was observed (Fig. 2C, D; *P* < 0.001 and *P* < 0.05, respectively). Mice with an induced GBA deficiency in the white blood cell lineage showed, upon treatment with GCS inhibitor Genz-112638 (eliglustat tartrate, Genzyme), partial reduction in plasma GlcCer (37) and an almost statistically significant reduction of elevated plasma GlcChol (Fig. 2E, *P* = 0.07). An increase in GlcChol was also observed in liver (Fig. 2F, *P* < 0.001), spleen, and bone marrow (see supplementary Fig. 1) of mice with induced GD. Correction of GBA deficiency by lentiviral gene therapy under the control of different promoters led, in all cases, to a concomitant reduction of GlcChol in these tissues (Fig. 2F).

### In vitro transglycosylation by β-glucosidases

The enzymes GBA and GBA2 were both found able to hydrolyze GlcChol at conditions optimal for degradation of 4MU-Glc (supplementary Table 1).

The findings on GlcChol levels in WT, GBA-deficient, and GBA2-deficient mice prompted us to study the ability of the three β-glucosidases, GBA, GBA2, and GBA3, to generate glucosylated cholesterol by transglucosylation. We first studied this ability in vitro and reproduced the assay of Akiyama and coworkers (11) using 25-NBD-cholesterol as acceptor and detection of 25-NBD-glucosyl-cholesterol formation by TLC and fluorescence scanning. As the source of enzyme, we used rGBA and individually overexpressed GBA2, GBA3, and GCS in COS-7 cells. Overexpression of enzymes was checked by measuring enzymatic activity with appropriate assays (not shown).

**Fig. 3.**
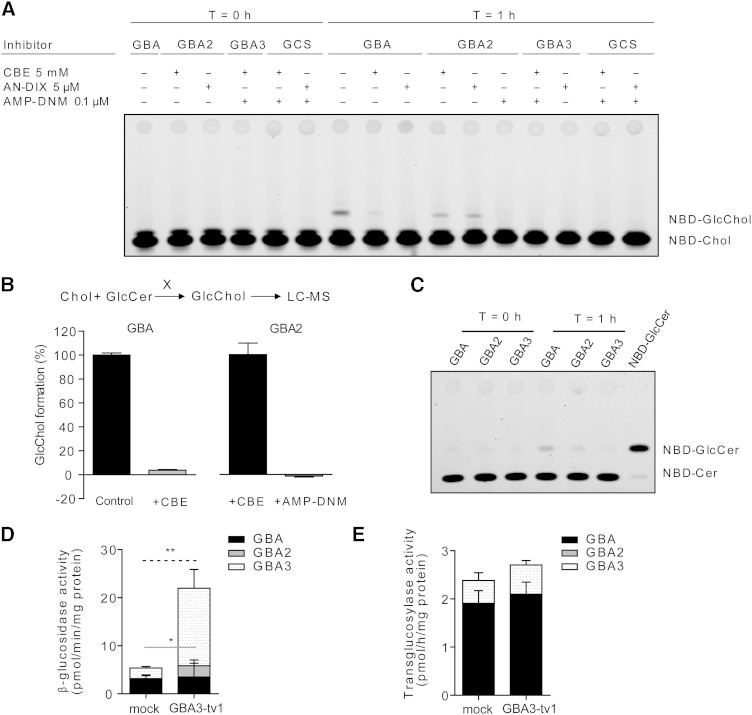
In vitro transglucosylation of 25-NBD-GlcChol by GBA and GBA2. A: rGBA and lysates of cells with overexpression of GBA2, GBA3, and GCS were incubated for 0 and 1 h with 25-NBD-cholesterol in the presence of C18:1-GlcCer as donor, and in the absence or presence of the respective specific β-glucosidase inhibitors: CBE (GBA), AMP-DNM (GBA2), and anDIX (GBA3) (26). Formation of 25-NBD-GlcChol was detected by HPTLC and fluorescence scanning. B: rGBA (in absence or presence of CBE) and lysates of cells with overexpression of GBA2 (in presence of CBE and in absence or presence of AMP-DNM) were incubated for 0 and 1 h with cholesterol in the presence of C18:1-GlcCer as donor. Formation of GlcChol was detected by LC-MS. The percentage of inhibition of GlcChol formation by the respective inhibitors is shown. Data presented as mean ± SD. C: rGBA and lysates of cells with overexpression of GBA2 and GBA3 were incubated for 0 and 1 h with GlcChol in the presence of NBD-Cer. Formation of NBD-GlcCer was detected by HPTLC and fluorescence scanning. D: β-Glucosidase activity in lysates of CHO-K1 cells overexpressing GBA3. The activity was measured in the absence or presence of the specific β-glucosidase inhibitors: 0.5 mM CBE and/or 0.3 mM NB-DGJ. CBE- and NB-DGJ-sensitive activities were defined as activities derived from GBA (black box) and GBA2 (gray box), respectively. CBE- and NB-DGJ-insensitive activity was defined as activity derived from GBA3 (dotted box). Mock represents the cells transfected with empty vector. E: Transglucosylase activity in lysates of cells with overexpression of GBA3. Data (n = 3, mean ± SD) were analyzed using an unpaired *t*-test. *P < 0.05 and **P < 0.01. Black, gray, and dotted lines show the significant difference of the activity derived from GBA, GBA2, and GBA3, respectively.

Recombinant enzyme and COS-7 cell lysates were incubated with natural GlcCer (C18:1-GlcCer) as donor and 25-NBD-cholesterol as acceptor. Following incubation at optimal conditions for each enzyme with inclusion of UDP-Glc for GCS (see Materials and Methods section), lipids were extracted and subjected to HPTLC. As shown in **Fig. 3A**, rGBA and cell lysates with overexpressed GBA2 generated an additional fluorescent lipid coinciding with 25-NBD-cholesterol-glucoside. Inhibition of GBA with CBE or GBA2 with AMP-DNM prevented formation of 25-NBD-cholesterol-glucoside (Fig. 3A). Transglucosylation was hardly observed for cell lysates with overexpressed GBA3 and GCS (Fig. 3A), recapitulating the findings by Akiyama and coworkers concerning GCS (10). We repeated the experiment using natural cholesterol as acceptor and determined the levels of formed GlcChol by LC-MS (Fig. 3B). Again, GlcChol formation occurred in the presence of GBA (inhibitable by CBE) and in cell lysates with high GBA2 (inhibitable by AMP-DNM) (Fig. 3B). Lysates of cells overexpressing GBA3 and GCS showed no additional GlcChol formation (supplementary Fig. 2). The pH optimum of GBA and GBA2 to generate GlcChol was next determined (see supplementary Fig. 3). In the case of GBA, optimal activity was seen between pH 4.5 and 5.5 and for GBA2 between pH 6.0 and 7.0. We investigated, with the same assay, whether GlcSph, GalCer, and GalSph also act as glycose donors in the transglycosylation catalyzed by GBA or GBA2 (see supplementary Fig. 3). This was not observed.

We next studied whether natural GlcChol (100 μM) can also act as donor in transglucosylation by incubating rGBA and lysates of cells overexpressing GBA2 or GBA3 in the presence of NBD-ceramide (40 μM) as acceptor. Lysates of cells overexpressing GBA2 and rGBA showed formation of fluorescent NBD-GlcCer (Fig. 3C). This was not observed for lysates with overexpressed GBA3 (Fig. 3C). Transglucosylation by both GBA and GBA2 occurs as an equilibrium reaction in which the glucose moiety is reversibly exchanged between cholesterol and ceramide.

We investigated in more detail potential transglucosylase activity of human GBA3 overexpressed in CHO-K1 cells. Overexpression of GBA3 increased β-glucosidase activity (Fig. 3D). As shown in Fig. 3E, overexpression of GBA3 did not affect transglucosylase activity from natural GlcCer (C16:0-GlcCer) to 25-NBD-cholesterol despite the long reaction time (20 h). Almost the same results were observed in cell lysates incubated with natural GlcCer (C18:0-GlcCer or C24:1-GlcCer) as donor (data not shown).

Molecular docking of GlcChol in the GBA crystal structure was performed (see supplementary Fig. 4). The ligand GlcChol was built and regularized with ligand and superimposed on the bicyclic nojirimycin analog ligand that was crystallized in complex with GBA (protein data bank code 2XWE) using the program, Coot (see supplementary Methods). GlcChol was found to be positioned such that the glucosidic bond is accessible to the catalytic residues Glu235 and Glu340. The cholesterol moiety concomitantly interacts with aromatic side chains.

### Metabolism of GlcChol in cultured COS-7 cells

**Fig. 4.**
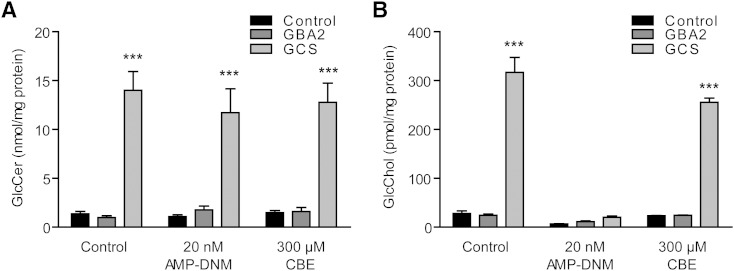
GlcChol in COS-7 cells manipulated in GSL-metabolizing enzymes. A: GlcCer (nanomoles per milligram protein) in COS-7 cells without overexpression of enzymes (control), overexpressed GBA2, and GCS. Cells were incubated for 2 days with the indicated inhibitors of GBA2 (AMP-DNM) and GBA (CBE). B: GlcChol (picomoles per milligram protein) in same cells. Data (n = 4, mean ± SD) were analyzed using an unpaired *t*-test. ***P < 0.001.

Next we studied factors influencing the formation of GlcChol content in cultured green monkey kidney COS-7 cells. We first studied the impact of overexpressed GBA2 and GCS. **Figure 4** shows the effect on cellular GlcChol and GlcCer levels. Only overexpression of GCS led to increased levels of GlcCer (Fig. 4A). GlcChol was not changed by overexpression of GBA2, but overexpression of GCS caused a 12-fold increase in this lipid. Importantly, inhibition of GBA2 activity with low nanomolar AMP-DNM (38) resulted in reduced cellular GlcChol. Even in cells with overexpressed GCS, the elevation in GlcChol was prevented (Fig. 4B). In contrast, inhibition of GBA with CBE hardly diminished the increased GlcChol level in cells with overexpressed GCS. These findings suggest that GCS does not generate GlcChol itself, but is required to generate sufficient GlcCer to be used as donor in formation of GlcChol by transglucosylation. This transglucosylation in COS-7 cells is particularly mediated by GBA2, and not GBA. The latter notion is consistent with the finding that GBA2 deficiency in mice, and not that of GBA, is accompanied with reduced GlcChol levels of tissues.

### GlcChol in NPC mice and U18666A-treated cells

**Fig. 5.**
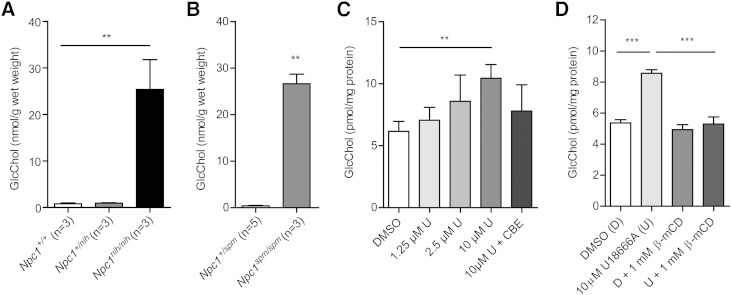
GlcChol abnormalities in NPC. A: GlcChol (nanomoles per gram wet weight) in liver of BALB/c *Npc1*^+/+^, *Npc1*^+/nih^ and *Npc1*^nih/nih^ male mice at 80 days of age (n = 3, mean ± SD). B: GlcChol (nanomoles per gram wet weight) in liver of C57BLKS *Npc1*^+/spm^ and *Npc1*^spm/spm^ male mice at 80 days of age (n = 3–5, mean ± SD). C: GlcChol (picomoles per milligram protein) in RAW264.7 cells incubated with the indicated concentration of U18666A for 1 day in the absence and presence of CBE-inhibiting GBA (n = 3, mean ± SD). D: GlcChol picomoles per milligram protein) in RAW264.7 cells incubated with 10 μM U18666A for 8 h and in the subsequent absence or presence of 1 mM β-methyl-cyclodextrin (β-mCD) reducing intralysosomal cholesterol for 18 h (n = 3, mean ± SD). Data were analyzed using an unpaired *t*-test. **P < 0.01, and ***P < 0.001.

In NPC disease, cholesterol accumulates prominently in lysosomes as the result of impaired export from the compartment due to defects in either *Npc1* or *Npc2*. In the livers of spontaneous *Npc1^nih/nih^* (29, 39, 40) and *Npc1^spm/spm^* mice (30, 41), we observed a remarkable 25-fold increase in GlcChol content (**Fig. 5A, B**). The identity of the measured glucosylated sterol was examined by digestion with rGBA. While more than 90% of the GlcChol in the livers of WT mice was digested to cholesterol, in the case of material from NPC mice, this was only around 70% (not shown). Based on this finding, it seems likely that part of the elevated compound with *m/z* 566.6 > 369.4 in NPC liver consists of cholesterol molecules modified differently with sugar, indistinguishable from glucosyl-β-D-cholesterol with the LC-MS method.

To experimentally substantiate the observations made for GlcChol in the livers of NPC mice, we induced impaired cholesterol export from lysosomes in murine macrophage RAW264.7 cells by exposure to U18666A (42). Following lysosomal cholesterol accumulation, cells showed elevated GlcChol. Concomitant inhibition of lysosomal GBA by CBE prevented the increase of GlcChol in U18666A-exposed cells (Fig. 5C). Formation of excessive GlcChol was also prevented by the presence of 1 mM β-methyl-cyclodextrin, an agent known to reduce intralysosomal cholesterol in NPC cells (43). This indicates that during extreme intralysosomal accumulation of cholesterol, GBA actively generates GlcChol. In normal lysosomes GBA most likely largely degrades the glucosylated sterol.

### GlcChol in NPC and GD patients

**Fig. 6.**
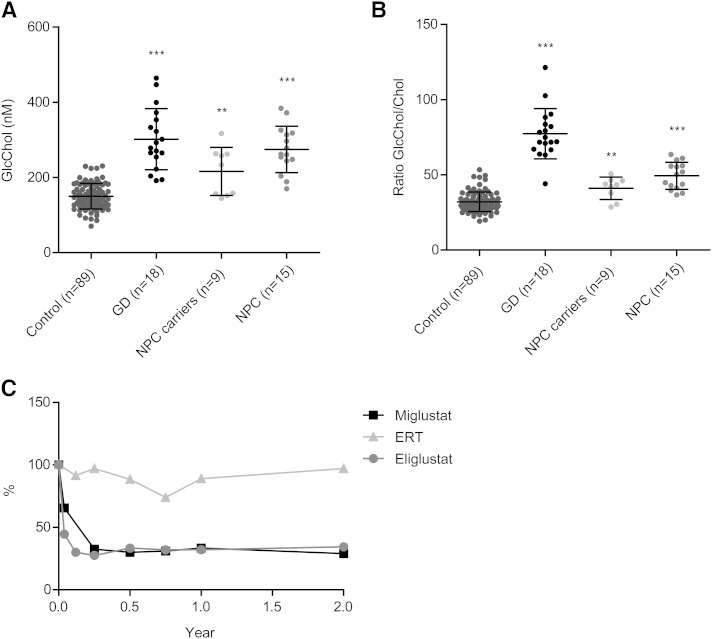
Plasma GlcChol in lysosomal storage disease patients and healthy individuals. A: Plasma GlcChol (nanomolar) in healthy individuals, type 1 GD patients, NPC carriers, and NPC patients. B: Ratio of GlcChol/cholesterol (Chol) in plasma of healthy individuals, type 1 GD patients, NPC carriers, and NPC patients. C: Percentage reduction in plasma GlcChol of matched type 1 GD patients following eliglustat, miglustat, and ERT treatment compared with pretreatment values. Data were analyzed using the Mann-Whitney U test. **P < 0.01, and ***P < 0.001.

Finally, we determined GlcChol levels in the plasma of untreated symptomatic type 1 GD patients, as well as in NPC patients, carriers, and healthy controls. As shown in **Fig. 6A**, GlcChol tends to be increased in the plasma of symptomatic GD patients and less prominently in that of NPC patients. The abnormalities in GD patients are more pronounced when plasma GlcChol is related to cholesterol (Fig. 6B). Investigation of plasma specimens of type 1 GD patients treated with the GCS inhibitor, eliglustat (Fig. 6C), showed a prominent reduction upon inhibition of GSL synthesis. Treatment of matched patients with the weaker GCS inhibitor, miglustat (Zavesca^®^, Actelion) also led to a reduction of GlcChol, albeit more slowly (Fig. 6C). Of note, treatment of matched patients with rGBA Cerezyme (ERT) did not reduce GlcChol to the same extent, despite impressive clinical improvements in these patients (Fig. 6C) (B. E. Smid et al., unpublished observations).

## DISCUSSION

Recently, Akiyama and colleagues (11) demonstrated that GBA, either pure recombinant protein or enzyme in fibroblast lysates, can generate GlcChol by transglucosylation of cholesterol when provided with GlcCer as donor. The physiological significance of glucosylation of cholesterol is hypothetically great because it renders a molecule far more water soluble. To establish the natural occurrence of GlcChol in mammals, we first needed to develop a quantitative and sensitive assay for quantification of GlcChol in plasma, cells, and tissues. The method developed by us makes use of newly synthesized ^13^C_6_-GlcChol as internal standard. Regarding extraction efficiency, chromatographic behavior, and ionization characteristics, the natural and isotope-labeled compounds are identical, so no correction for the sample matrix (ion suppression or ion enhancement) is required.

With sensitive quantification of GlcChol in place, we next observed that almost all tissues of mice show GlcChol. The relative high amount of the glucosylated sterol in the thymus, several nanomoles per gram wet weight, is striking and deserves special attention in view of noted abnormalities in NKT and B-cells in GBA-deficient GD patients (44). It has been speculated by Mistry and colleagues that elevated GlcCer or GlcSph, via binding to CD1, may cause this phenomenon (44). It will now be of interest to study whether GlcChol interacts with CD1 because abnormalities in concentration of this lipid in GBA-deficient GD patients are likely.

Indeed, we recapitulated the finding that GBA is able to form GlcChol by transglucosylation of cholesterol, at least in vitro. Artificial β-glucosides, like 4MU-Glc, may act as donor in this reaction in vitro, as well as natural GlcCer. GlcChol is, on the other hand, also an excellent substrate for hydrolysis by GBA. Our findings suggest that GBA normally lowers GlcChol levels. GBA-deficient LIMP2 KO mice show modestly elevated GlcChol in several tissues and the same is observed in GBA^null/flox^ mice with induced type 1 GD (32). Finally, plasma GlcChol is elevated in symptomatic type 1 GD patients. The actual role played by GBA in GlcChol metabolism, degradation versus synthesis, might be highly dependent on local concentrations of donors (GlcCer and GlcChol) and acceptors (ceramide and cholesterol) in the transglucosylation equilibrium of the enzyme. The importance of this is suggested by some observations made in the course of our investigation. In the first place, high intralysosomal cholesterol concentrations appear to favor formation of GlcChol by GBA. This is indicated by the 25-fold elevated GlcChol in the liver of two different models of NPC disease in mice. In accordance with this, induction of lysosomal cholesterol accumulation with U18666A in cells causes a rapid increase in GlcChol, which was noted to be abolished by inactivation of GBA. Consequently, under normal conditions, GBA seems to promote GlcChol degradation, but under excess cholesterol accumulation in lysosomal membrane, such as that found in NPC, the equilibrium of the metabolism is altered to favor GlcChol formation by GBA.

We observed that the nonlysosomal β-glucosidase, GBA2, can also generate GlcChol through transglucosylation in vitro. Again, GlcCer was found to be an excellent donor for this reaction. GBA2 is equally able to degrade GlcChol. Our finding of reduced GlcChol levels in GBA2 KO mice suggests, but does not entirely prove, that GBA2 in vivo contributes to the presence of GlcChol. The enzyme GBA2 is located differently in cells from lysosomal GBA, with its catalytic pocket exposed to the cytoplasmic leaflet of membranes. It likely encounters different concentrations of GlcCer and cholesterol than GBA, given its location close to the cellular sites of de novo synthesis of these lipids. To maximally form GlcChol through transglucosylation, high concentrations of GlcCer as donor and high concentrations of cholesterol as acceptor are optimal. Vice versa, low concentrations of GlcCer and cholesterol will reduce net GlcChol formation. This consideration holds equally for GBA2 and GBA. Fluctuations in sterols and sphingolipids conceivably occur in cells, for example, after uptake of cholesterol-rich lipoproteins or upon release of ceramide from sphingomyelin. The ability to maintain some equilibrium between (glucosylated) sphingolipids and sterols by transglucosylating β-glucosidases may have beneficial buffering effects for cells. Of interest in this respect is our finding that inhibition of GCS leads to reduction of GlcChol in cultured cells, plasma of mice, and plasma of GD patients. This strongly suggests that the availability of GlcCer is an important driver of formation of GlcChol through transglucosylation. Because the β-glucosidases, GBA and GBA2, also hydrolyze GlcCer, and thus tend to lower its concentration, the exquisite balance of various GlcCer-metabolizing enzymes and local cholesterol concentrations will determine GlcChol formation in subcellular compartments.

GlcChol is far more water soluble than cholesterol and, therefore, more suited for transport. The relatively low steady concentration of GlcChol does not exclude a vital role as intermediate in a transport pathway. Tentatively, water soluble GlcChol formed by transglucosylation at one cellular site would be transported and reconverted at the destination site back to more inert cholesterol. The combination of the two enzymes, GBA and GBA2, could provide such a mechanism without need for ATP. Of interest, in view of this speculation, is that LIMP2, the membrane protein interacting with GBA, has recently been shown to have a cholesterol binding site and potentially even a tunnel/channel function (45).

A final consideration concerns the possible pathophysiological consequences of disturbed GlcChol metabolism. It seems likely that in individuals with abnormal GlcCer metabolism, as for example GBA-deficient GD patients, secondary abnormalities in GlcChol occur. Future research will need to reveal whether such abnormalities in GlcChol or in other glucosylated metabolites contribute to particular symptoms associated with GD. In conclusion, GlcCer has earlier been identified as an important structural membrane component and intermediate in GSL biosynthesis. In addition, it is known to act as an important sink for pro-apoptotic ceramide (46, 47). Our study suggests that GlcCer may furthermore act as glucosyl-donor in the formation of GlcChol via transglycosylation.

## Supplementary Material

Supplemental Data
