# Supplementary material for: Glucosylated cholesterol in mammalian cells and tissues: formation and degradation by multiple cellular β-glucosidases
Source: J Lipid Res. 2016 Mar;57(3):451–63. doi: 10.1194/jlr.M064923 (PMC4766994; doi:10.1194/jlr.M064923)
Supplement: Supplemental Data [file supp_57_3_451__index.html]

Glucosylated cholesterol in mammalian cells and tissues: formation and degradation by multiple cellular β-glucosidases — Glucosylated cholesterol in mammalian cells and tissues: formation and degradation by multiple cellular β-glucosidases — Supplemental Data 

# Glucosylated cholesterol in mammalian cells and tissues: formation and degradation by multiple cellular β-glucosidases

## Supplemental Data

- Supplemental Information (.pdf, 467 KB) - Supplemental information contains: Supplemental Methods, Supplemental Table 1 and Supplemental Figures 1-4.
