# Supplementary material for: Glucosylated cholesterol in mammalian cells and tissues: formation and degradation by multiple cellular β-glucosidases
Source: J Lipid Res. 2016 Mar;57(3):451–63. doi: 10.1194/jlr.M064923 (PMC4766994; doi:10.1194/jlr.M064923)

Supplemental Information

Supplemental Methods

Reagents C18-GlcSph (D-glucosyl-β1-1′-D-*erythro*-sphingosine), C18-GalCer (D-galactosyl-β1-1′ N-palmitoyl-D-*erythro*-sphingosine) and GalSph (D-galactocosyl-β1-1′-D-*erythro*-sphingosine) were obtained from Avanti Polar Lipids (Alabaster, USA).

Molecular Modeling The ligand GlcChol was build and regularized with ligand (1) and superimposed on the bicyclic nojirimycin analogue ligand that was crystallized in complex with GBA (pdb code 2XWE) (2) using the program coot (3). Supplementary Figure 4 shows the resulting model of GBA complexed with GlcChol.

Supplemental References

1. Lebedev, A. A., P. Young, M. N. Isupov, O. V Moroz, A. A. Vagin, and G. N. Murshudov. 2012. JLigand: a graphical tool for the CCP4 template-restraint library. *Acta Crystallogr. D. Biol. Crystallogr.* **68**: 431–40.
2. Brumshtein, B., M. Aguilar-Moncayo, J. M. Benito, J. M. García Fernandez, I. Silman, Y. Shaaltiel, D. Aviezer, J. L. Sussman, A. H. Futerman, and C. Ortiz Mellet. 2011. Cyclodextrin-mediated crystallization of acid β-glucosidase in complex with amphiphilic bicyclic nojirimycin analogues. *Org. Biomol. Chem.* **9**: 4160–7.
3. McNicholas, S., E. Potterton, K. S. Wilson, and M. E. M. Noble. 2011. Presenting your structures: the CCP4mg molecular-graphics software. *Acta Crystallogr. D. Biol. Crystallogr.* **67**: 386–94.

Supplemental Table 1. Degradation of GlcChol by GBA and GBA2.

	Input: nmol 4MU-β-Glc hydrolysis per mL/min	Percentage GlcChol digestion (200 pmole)
rGBA	1000	99 %
GBA2	0.04	77 %

Supplemental Figure Legends

Supplemental Figure 1. Increased GlcChol in spleen and bone marrow of mice with induced type 1 GD.

A. Spleen and B. bone marrow GlcChol in *wt* mice, type 1 GD induced mice untreated, type 1 GD treated with lentiviral GBA cDNA gene therapy with macrophage specific promotor (CD68), ubiquitously expressed human phosphoglycerate kinase (PGK) promotor or gammaretroviral vector with the viral promoter spleen focus forming virus (SFFV) promotor. Data were analyzed using an unpaired t-test. * $P < 0.05$, ** $P < 0.01$ and *** $P < 0.001$.

Supplemental Figure 2. *In vitro* formation of GlcChol by different β-glucosidases.

Recombinant rGBA and lysates of cells with overexpression of GBA2, GBA3 or GCS were incubated for 0 and 1 h with cholesterol in the presence of C18:1-GlcCer as donor. Formation of GlcChol (nmol/L*h) was detected by LC-MS. Inhibition of GlcChol formation by the respective β-glucosidase inhibitors – CBE (GBA), AMP-DNM (GBA2) and anDIX (GBA3) – is shown.

Supplemental Figure 3. *In vitro* formation of GlcChol: pH dependence and donor preference.

A. rGBA (in McIlvaine buffer 0.15 M, 0.2% taurocholate and 0.1% Triton X-100) and lysates of cells overexpressing GBA2 (in McIlvaine buffer 0.15 M) were incubated for 1 h with 25-NBD-cholesterol in the presence of C18:1-GlcCer as donor at different pHs. B. rGBA (in McIlvaine buffer 0.15M pH 5.2, 0.2% taurocholate and 0.1% Triton X-100) and lysates of cells overexpressing GBA2 (in McIlvaine buffer 0.15 M pH 5.8) were incubated for 1 h with 25-NBD-cholesterol in the presence of different donors (100 μM): 4-MU-glucopyranoside, C18:1-GlcCer, C18-GlcSph, C18-GalCer and C18-GalSph.

Supplemental Figure 4. Molecular docking of GlcChol in GBA crystal structure 2XWE. GlcChol docked on GBA crystal structure 2XWE. GlcChol is shown in green with its oxygen atoms in red. GBA is shown in blue (and gold) with the catalytic residues Glu235 and Glu340 labeled. Side chains that are within 5 Å of GlcChol are also displayed.

Supplemental Figure 1. Increased GlcChol in spleen and bone marrow of mice with induced type 1 GD.

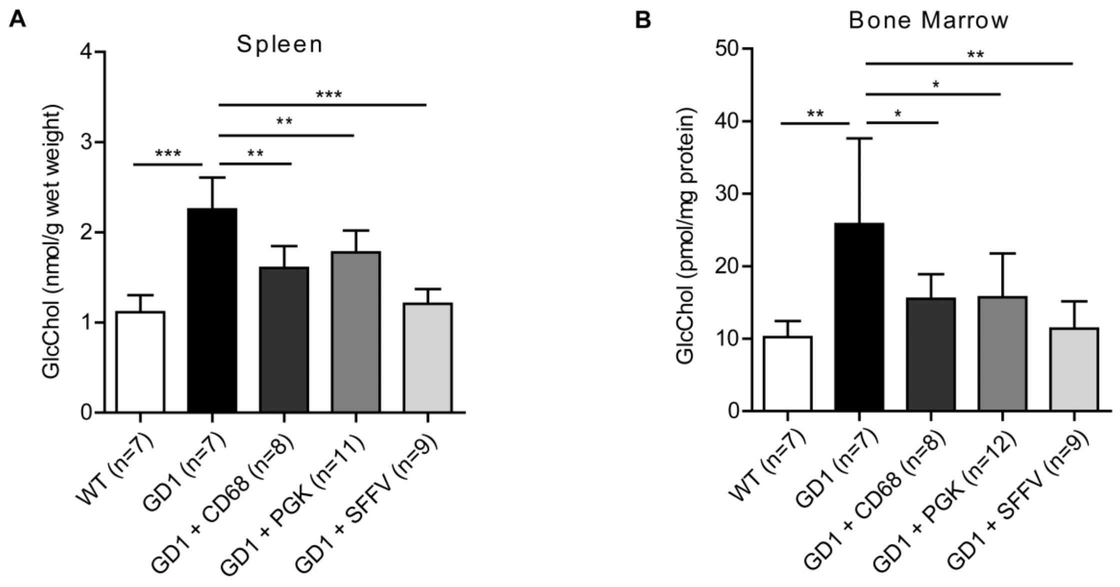

Supplemental Figure 2. ***In vitro*** **formation of GlcChol by different β-glucosidases.**

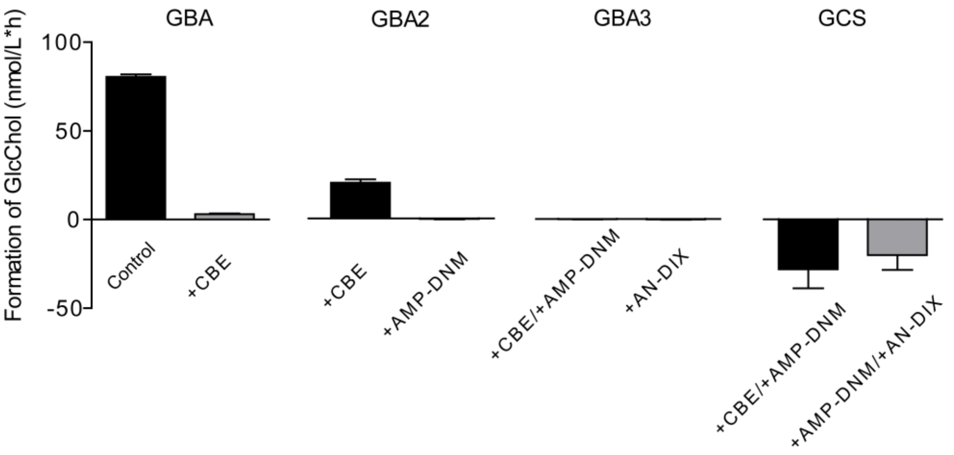

Supplemental Figure 3. *In vitro* formation of GlcChol: pH and substrate preference.

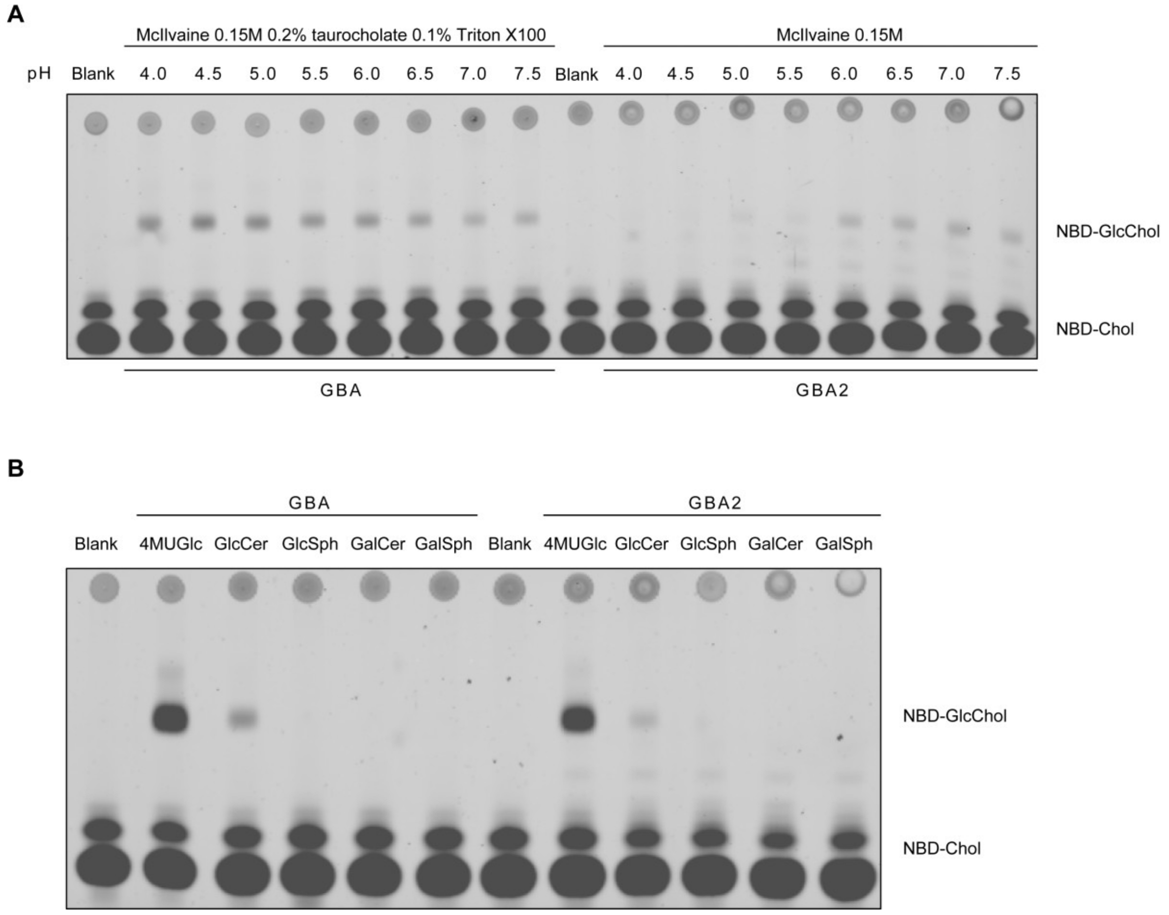

Supplemental Figure 4. Molecular docking of GlcChol in GBA crystal structure 2XWE.

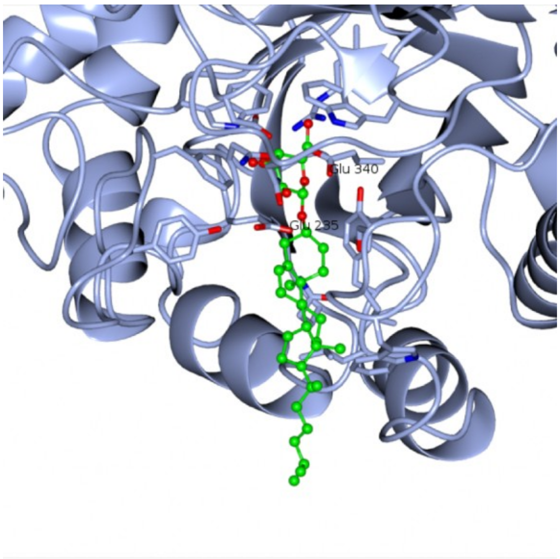

Supplement: Supplemental Data [file 10.1194_M064923_jlr.M064923-1.pdf]
